# A Highly Sensitive and Selective Colorimetric Hg^2+^ Ion Probe Using Gold Nanoparticles Functionalized with Polyethyleneimine

**DOI:** 10.1155/2018/1206913

**Published:** 2018-02-05

**Authors:** Kyung Min Kim, Yun-Sik Nam, Yeonhee Lee, Kang-Bong Lee

**Affiliations:** ^1^Green City Technology Institute, Korea Institute of Science and Technology, Hwarang-ro 14 gil 5, Seoul 02792, Republic of Korea; ^2^Department of Chemistry, Korea University, Anam-ro, Seongbuk-gu, P.O. Box 145, Seoul 136-701, Republic of Korea; ^3^Advanced Analysis Center, Korea Institute of Science and Technology, Hwarang-ro 14 gil 5, Seoul 02792, Republic of Korea

## Abstract

A highly sensitive and selective colorimetric assay for the detection of Hg^2+^ ions was developed using gold nanoparticles (AuNPs) conjugated with polyethyleneimine (PEI). The Hg^2+^ ion coordinates with PEI, decreasing the interparticle distance and inducing aggregation. Time-of-flight secondary ion mass spectrometry showed that the Hg^2+^ ion was bound to the nitrogen atoms of the PEI in a bidentate manner (N–Hg^2+^–N), which resulted in a significant color change from light red to violet due to aggregation. Using this PEI-AuNP probe, determination of Hg^2+^ ion can be achieved by the naked eye and spectrophotometric methods. Pronounced color change of the PEI-AuNPs in the presence of Hg^2+^ was optimized at pH 7.0, 50°C, and 300 mM·NaCl concentration. The absorption intensity ratio (*A*_700_/*A*_514_) was correlated with the Hg^2+^ concentration in the linear range of 0.003–5.0 *μ*M. The limits of detection were measured to be 1.72, 1.80, 2.00, and 1.95 nM for tap water, pond water, tuna fish, and bovine serum, respectively. Owing to its facile and sensitive nature, this assay method for Hg^2+^ ions can be applied to the analysis of water and biological samples.

## 1. Introduction

Mercury ion (Hg^2+^) is ubiquitously distributed in the environment, and it is considered to be one of the major environmental pollutants to be widely used in industry, agriculture, and medicine. It is nonessential and toxic to the human body. Hg^2+^ is considered to be one of the major environmental pollutants to be widely used in industry, agriculture, and medicine. This mercury ion exists in inorganic and organic mercury ions. Upon entering the body, inorganic mercury ions are accumulated mainly in the kidneys and give rise to vomiting and diarrhea, followed by hypovolemic shock, oliguric renal failure, and possibly death [1–4]. Organic mercury ions such as methylmercuric (MeHg^+^), ethylmercuric (EtHg^+^), and phenylmercuric (PhHg^+^) ions can also cause injuries at the central nervous system and lead to paresthesias, headaches, ataxia, dysarthria, visual field constriction, blindness, and hearing impairment [5–7]. Therefore, detection of mercury ion in various sample matrices has been an urgent issue.

Several analytical techniques, such as direct mercury analyzer (DMA) [8], ion chromatography (IC) [9], and high performance liquid chromatography (HPLC) [10, 11], have been utilized to detect mercury ions. However, these ways generally require complicated sample pretreatment process, skillful technicians, and sophisticated instrumentations. Therefore, the low-cost and facile analytical method for selective detection of mercury ions remains to be a challenge for analytical chemists.

Various nanoparticle assays for a simple detection of Hg^2+^ ions have recently been investigated using gold nanorods (AuNRs) [12], carbon nanoparticles (CNPs) [13], silver nanoparticles (AgNPs) [14], silver nanoprisms (AgNPRs) [15], and AuNPs [16–18]. These colorimetric methods are especially promising in the analysis of Hg^2+^ with their naked eye or UV-Vis applications, due to their high extinction coefficients and the interparticle distance-dependent optical properties.

Polyethyleneimine (PEI) was applied to use a chemical functionalizer as a harmless gene delivery mediator, templates, stabilizers, and molecular gum to arrange metal nanoparticles [19–22]. Various amine groups could give sufficient active sites for strong combining capability, and these characteristics of PEI can be useful to control the selectivity of different ions. For its application, AuNPs conjugated with PEI (PEI-AuNPs) have been utilized as delivery of drug and gene in breast cancer therapy [23].

This study showed that PEI-AuNPs were aggregated with inorganic and organic mercury ions, and these ions induced the definite color change of AuNPs selectively among other diverse ions. Dispersed and aggregated AuNPs were characterized by ultraviolet-visible spectroscopy (UV-Vis), high-resolution transmission electron microscopy (HR-TEM), and dynamic light scattering (DLS) upon addition of Hg^2+^. Hg^2+^ ion binding sites on the surface of PEI-AuNPs were elucidated by ^13^C nuclear magnetic spectroscopy (^13^C NMR), X-ray photoelectron spectroscopy (XPS), and time-of-flight secondary ion mass spectrometry (TOF-SIMS) [24, 25]. The interference effects were tested in the presence of other metal ions and anions. Also, PEI-AuNP assay method for detection of Hg^2+^ ion was optimized in terms of pH, temperature, and salt condition.

This present assay using PEI-AuNP was very simple, cost-efficient, and allowed for the on-site detection of Hg^2+^ in real time. The limit of detection (LOD) was ∼2 nM in various samples. Therefore, this technique could be utilized to monitor Hg^2+^ ions in a wide range of practical samples.

## 2. Experimental

### 2.1. Materials

Gold (III) chloride trihydrate (HAuCl_4_·3H_2_O), PEI, methylmercuric chloride, and phenylmercuric chloride were sourced from Sigma-Aldrich (St. Louis, MO, USA). Ethylmercuric chloride was obtained from Chem Service (Tower Lane, West Chester, USA) Salts of NO_3_^−^, NO_2_^−^, PO_4_^3−^, SO_4_^2−^, Br^−^, Cl^−^, F^−^, Ca^2+^, Cd^2+^, Fe^3+^, Ba^2+^, Mn^2+^, Ga^3+^, Ti^4+^, Al^3+^, Mg^2+^, K^+^, Ge^4+^, Cr^3+^, Cu^2+^, Li^+^, As^3+^, Co^2+^, Sn^2+^, Pb^2+^, Hg^2+^, Ni^2+^, and Zn^2+^ were purchased from AccuStandard (New Haven, CT, USA). NaCl, HCl, and NaOH were purchased from Samchun Chemical (Gyeonggi-Do, Korea). Tap water was acquired from our laboratory and pond water was obtained from a pond at the Korea Institute of Science and Technology (KIST). Tuna fish was sourced from Dongwon (Seoul, Republic of Korea). Bovine serum was purchased from Sigma-Aldrich (St. Louis, MO, USA). Citrus leaf sample was obtained from Swan Leaf Pty Ltd (Perth, WA, Australia). Distilled water was obtained using a Milli-Q water purification system (Millipore, Bedford, MA, USA).

### 2.2. Preparation of PEI-AuNPs

PEI-AuNPs were synthesized as following literature procedures by mixing aqueous solutions of HAuCl_4_·3H_2_O (1 mL, 0.025 M) and PEI (7.8 mL, 9.74 mM), with subsequent reduction of HAuCl_4_ at ∼pH 7.0 [26]. The mixture was kept to react for three days at ambient temperature, and ∼10 nm PEI-AuNPs were produced.

### 2.3. Sample Preparation and Hg^2+^ Sensing Test Using PEI-AuNPs

The suspended particles in all water samples were removed by a syringe filter (0.20 *μ*m pore size) prior to analysis, and sample aliquots (9 mL) were mixed with a 500 *μ*M·Hg^2+^ solution (1 mL) to produce a 50 *μ*M·Hg^2+^ stock solution.

Tuna fish was obtained from a local supermarket. Its muscle tissues were crushed and dried on petri dishes overnight at 85°C. A small portion (ca. 0.3 g) was incubated in 2 mL HNO_3_ for 1 h before addition of 0.5 mL HClO_4_ (70%). Then, the samples were irradiated under a UV lamp for 3 h to convert all possibly contained organic mercury to inorganic mercury. Finally, the acid extracts were transferred to 50 mL volumetric flasks, and the volume was adjusted to 50 mL with Milli-Q water [27]. These samples were spiked with 100 *μ*g·mL^−1^ of Hg^2+^.

Stock solution of bovine serum was made to be 0.1% concentration in water. The suspensions were stirred and centrifuged alternately for 30 min and the solutions (9 mL) were blended with 1 mL of a 100 *μ*g·mL^−1^ Hg^2+^ solution.

0.9 g of citrus leaf was added to 5 mL concentrated HNO_3_ and heated for 2 h in the boiling water bath. After being cooled to room temperature, the samples were added with 2 mL of 30% H_2_O_2_, followed by heating for 1 h in the boiling water bath. Finally, the sample volume was made to 25 mL with double-distilled water. Before conducting experiments, the pH value of samples solution was neutralized by solid NaOH [28].

To evaluate the utility of our proposed method, Hg^2+^ concentrations added in tap, pond water, tuna fish, and bovine serum were measured with 1 mL of the PEI-AuNP solution, and followed by UV-Vis spectrophotometry. Those analytical results were confirmed with DMA.

### 2.4. Instrumentation

The absorption spectra were recorded by UV-Vis spectrophotometer (S-3100, Sinco, Seoul, Republic of Korea). UV-Vis spectra were acquired in the range of 300–800 nm by using 4 mm path length quartz cells. The pH measurements were conducted with an HI 2210 pH meter (Hanna Instruments, Woonsocket, RI, USA). The concentrations of Hg^2+^ ions in various samples were measured by DMA (DMA 80, Milestone, Italy). ^13^C NMR spectra were measured on an Avance III 400 MHz ^1^H NMR spectrometer (Bruker, Billerica, MA, USA). XPS analysis was performed using a PHI 5000 VersaProbe III instrument (ULVAC-PHI, Chigasaki, Japan). Mass spectra were measured using TOF-SIMS (TOF-SIMS 5, ION-TOF, Mȕnster, Germany). The size distributions of nanoparticles were recorded by a Zetasizer (Malvern Instruments Ltd., Worcestershire, UK). The images and sizes of PEI-AuNPs and their Hg^2+^-induced aggregates were measured on a micrograph using transmission electron microscope (TEM; CM30, Philips, NC, USA). TEM samples were obtained by settling the scattered AuNPs and evaporating the solvent.

## 3. Results and Discussion

### 3.1. Characterization of PEI-AuNPs and Their Complexes with Hg^2+^

**Figure 1:**
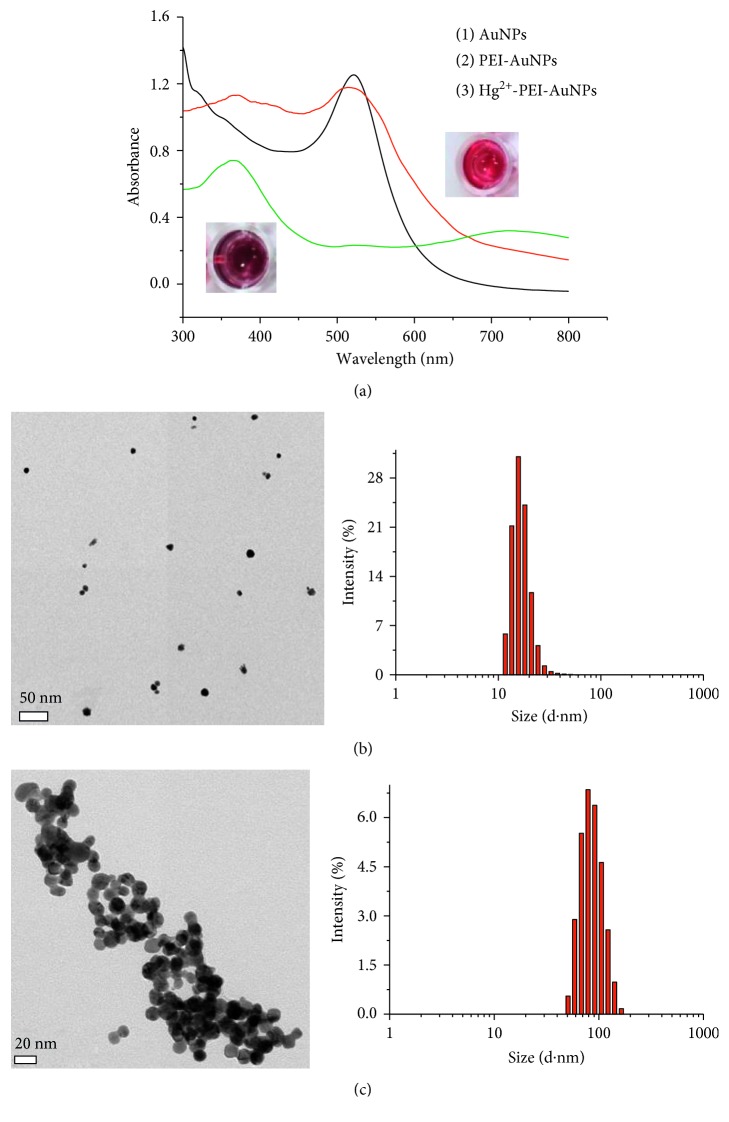
(a) UV-Vis absorption spectra of (1) AuNPs (black line), (2) PEI-AuNPs (red line), and (3) PEI-AuNPs conjugated with Hg^2+^ (green line). (b) TEM image of PEI-AuNPs (left) and the corresponding particle-size distribution histogram (right). (c) TEM image of PEI-AuNPs conjugated with Hg^2+^ (left) and particle-size distribution histogram (right).

PEI-AuNPs were prepared as ∼10 nm size as reducing HAuCl_4_ with amine group of PEI. The AuNPs were usually synthesized by the citrate reduction of HAuCl_4_, and their sizes were ∼33 nm as described in earlier studies [29], but AuNPs conjugated with PEI (PEI-AuNPs) under these conditions became much smaller. As a result, the mean AuNP size depended on the quantity and type of reducing agents, pH, temperature, and reaction time [30, 31]. A strong localized surface plasmon resonance (LSPR) peak of these label-free AuNPs appeared at ca. 514 nm in their UV-Vis spectrum, resulting in the red color of the corresponding solution. The size of AuNPs influenced to change their surface plasmon absorption maxima at 514 nm [32]. The sizes of PEI-AuNPs and Hg^2+^-PEI-AuNPs were distributed to be ∼15 and ∼75 nm, respectively, according to TEM images and Zetasizer measurements (Figures 1 and 1). The color of these PEI-AuNPs was similar to those of label-free AuNPs, but distinct color change occurred from red to dark violet upon addition of Hg^2+^ ions. UV-Vis absorption spectra for AuNP, PEI-AuNP, and Hg^2+^-PEI-AuNP solutions are demonstrated in Figure 1. Upon addition of Hg^2+^ ions, the strong absorption band of PEI-AuNPs at 514 nm was gradually shifted to 700 nm, and a new absorbance band concomitantly increased in intensity upon addition of Hg^2+^ ions (Figure 1). When PEI-AuNPs are aggregated, the conduction electrons near their surfaces become delocalized and are shared amongst neighboring particles. As a result, the surface plasmon resonance (SPR) shifts to lower energies, causing the shift of absorption and scattering peaks to longer wavelengths.

### 3.2. Selectivity of PEI-AuNPs for Hg^2+^ Ions and Related Interference Effects

**Figure 2:**
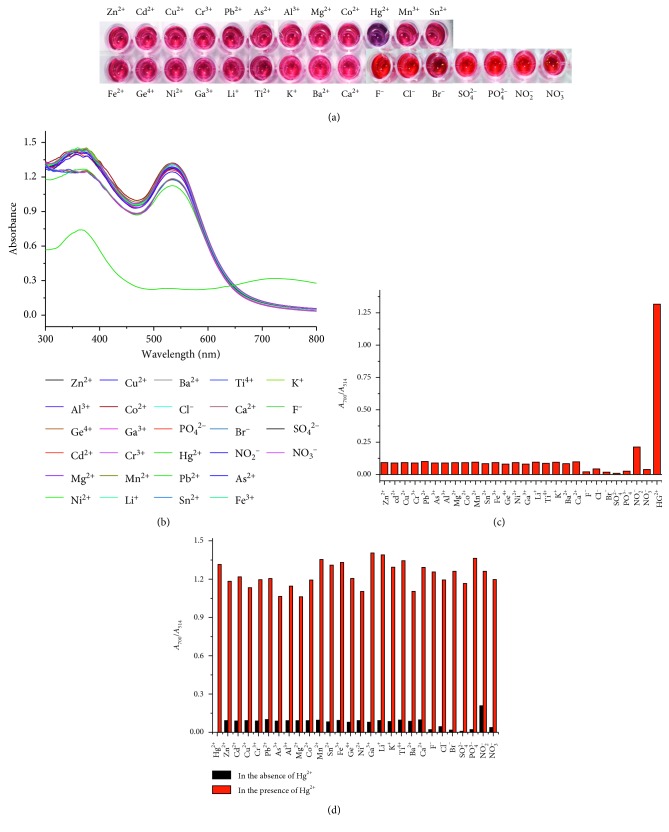
(a) Photographic images, (b) UV-Vis absorption spectra, (c) absorption ratios (*A*_700_/*A*_514_) of PEI-AuNPs with 5.0 *μ*M·Hg^2+^ ion and 50 *μ*M various ions at pH 7, 50°C, and 500 mM·NaCl concentration, and (d) absorption ratios (*A*_700_/*A*_514_) of PEI-AuNPs with 50 *μ*M various ions in the presence and absence of 5.0 *μ*M·Hg^2+^.

The selectivity for PEI-AuNP assay method was tested in 50 *μ*M·Hg^2+^ and various 500 *μ*M metal cations (Zn^2+^, Cd^2+^, Cu^2+^, Cr^3+^, Pb^2+^, As^3+^, Al^3+^, Mg^2+^, Co^2+^, Mn^2+^, Sn^2+^, Fe^3+^, Ge^4+^, Ni^2+^, Ga^3+^, Li^+^, Ti^4+^, K^+^, Ba^2+^, and Ca^2+^ ions) and anions (F^−^, Cl^−^, Br^−^, SO_4_^2−^, PO_4_^3−^, NO_2_^−^, and NO_3_^−^ ions). The interference by other 500 *μ*M numerous anions and cations for the selectivity of Hg^2+^ was further examined. Any metal cations and anions did not induce any color changes except Hg^2+^, as shown in Figure 2. UV-Vis absorption spectra of PEI-AuNPs solutions at pH 7, 50°C, and 300 mM·NaCl concentration were recorded in the presence of various metal cations and anions (Figure 2). The strong absorption band at 700 nm distinctly appeared for Hg^2+^, enabling to discriminate easily from different metal cations and anions. The absorbance ratios (*A*_700_/*A*_514_) of the PEI-AuNPs solution upon addition of each cation and anion were measured to test the selectivity for Hg^2+^ ion (Figure 2). The absorbance ratio of PEI-AuNPs solution in the presence of Hg^2+^ was ∼11 times greater than those in the presence of other ions. A high absorbance ratio of Hg^2+^-PEI-AuNPs was attributed to the aggregation of PEI-AuNPs, whereas a low absorbance ratio of PEI-AuNPs in the presence of other ions indicated to keep well-dispersed forms of PEI-AuNPs. Therefore, Hg^2+^ ion must be selectively coordinated with a specific site of PEI-AuNPs. The interference effect by other ions in the selectivity of Hg^2+^ toward PEI-AuNPs was tested in PEI-AuNP solutions upon addition of Hg^2+^ ions mixed with other ions. Other ions did not interfere with determination of Hg^2+^, even though their concentrations were ten times greater than that of Hg^2+^. No metal cations and anions except Hg^2+^ perturbed absorption bands at 514 and 700 nm (Figure 2).

### 3.3. Binding Sites of PEI to AuNPs and Hg^2+^ to PEI-AuNPs

XPS spectra for PEI-AuNPs and Hg^2+^-PEI-AuNPs were measured to confirm the binding site of Hg^2+^ ion to PEI-AuNPs (not shown) [33]. The high-resolution N 1 s signal in Hg^2+^-PEI-AuNPs at 406.2 eV showed the binding energy of Hg^2+^-N bonds [34]. Thus, it was found that Hg^2+^ ion must be bound to nitrogen atom of PEI.

**Figure 3:**
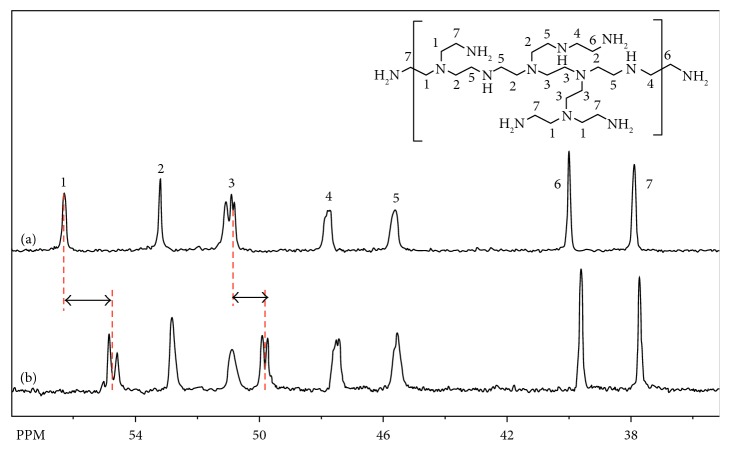
^13^C NMR spectrums for (a) PEI and (b) PEI-AuNPs in ^2^H_2_O at room temperature.

The binding site of Hg^2+^ to PEI was further examined with ^13^C NMR spectra for free PEI and PEI bound to Hg^2+^ (Hg^2+^-PEI) as a model of PEI-AuNPs and Hg^2+^-PEI-AuNPs (Figure 3). ^13^C NMR spectra showed that two CH_2_ peaks (peaks 1 and 3) resonating 56.5 ppm and 51.0 ppm in free PEI shifted significantly to 54.5 and 49.8 ppm, respectively, in comparison to those of Hg^2+^-PEI, as shown in Figure 3. The chemical shifts of other peaks changed a little, which indicated that Hg^2+^ ions must be coordinated to nitrogen atoms of tertiary amine in PEI [35, 36].

**Figure 4:**
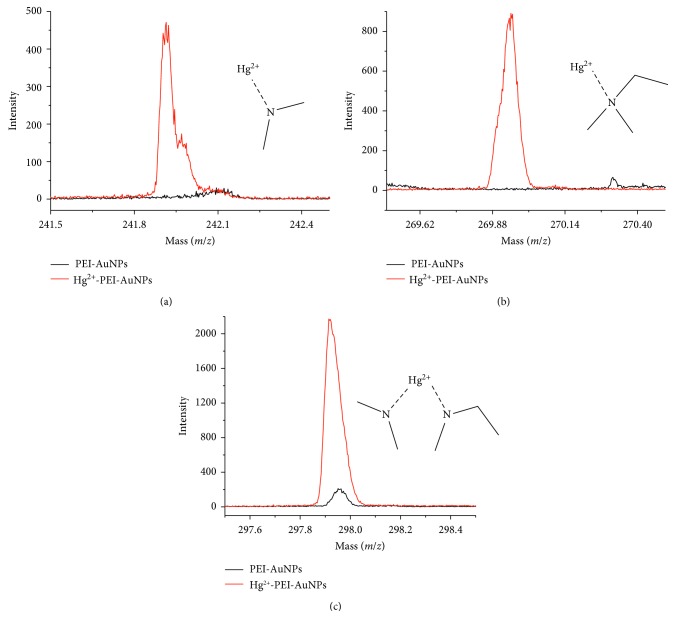
Mass peaks for (a) (CH_2_)_2_NHg^2+^ (*m*/*z*: 242), (b) CH_2_CH_2_(CH_2_)_2_NHg^2+^ (*m*/*z*: 270), and (c) (CH_2_)_2_NHg^2+^N(CH_2_)CH_2_CH_2_ (*m*/*z*: 298) fragments in TOF-SIMS spectra of PEI-AuNPs (black) and PEI-AuNPs-Hg^2+^ (red). These molecular fragments are expected based on PEI-AuNPs-Hg^2+^ structural elements in the zoomed circle in Scheme 1.

TOF-SIMS spectra for PEI-AuNPs and Hg^2+^-PEI-AuNPs showed an additional evidence for Hg^2+^ binding site to PEI-AuNPs (Figure 4). TOF-SIMS spectra of Hg^2+^-PEI-AuNPs provided an additional information for Hg^2+^ binding site to PEI-AuNPs. These MS spectra showed the molecular fragments of (CH_2_)_2_NHg^2+^ (*m*/*z*: 242), CH_2_CH_2_(CH_2_)_2_NHg^2+^ (*m*/*z*: 270), and (CH_2_)_2_NHg^2+^N(CH_2_)CH_2_CH_2_ (*m*/*z*: 298) in Hg^2+^-PEI-AuNPs (Figure 4). These molecular fragments did not appear for PEI-AuNPs, which indicated that Hg^2+^ must be coordinated with tertiary nitrogen atoms in PEI [37].

### 3.4. Optimum Conditions for PEI-AuNP Probe

**Figure 5:**
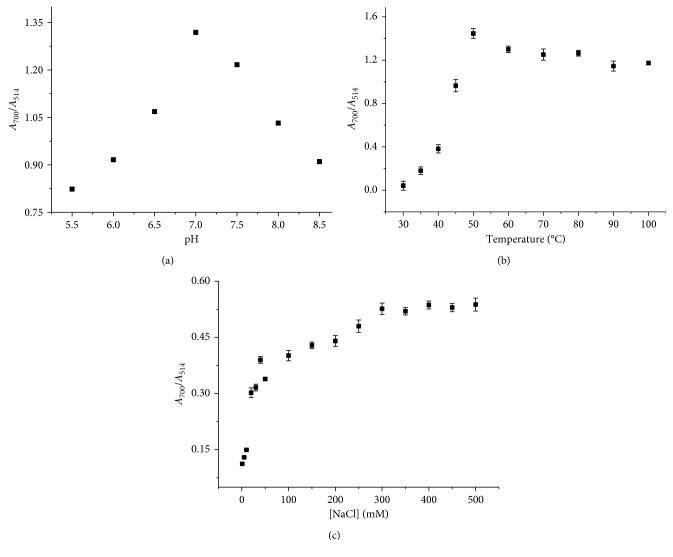
Absorption ratios (*A*_700_/*A*_514_) of Hg^2+^-PEI-AuNPs as a function of (a) pH, (b) temperature, and (c) concentration of NaCl.

To optimize the sensitivity of the PEI-AuNP probe for Hg^2+^, the probe was tested as functions of pH, temperature, salt concentration, PEI concentration, and reaction time. The absorbance ratios changed as a function of pH, and it was the highest at pH 7 (Figure 5). This optimum pH of the PEI-AuNP probe must be something to do with pKa of tertiary amine and the conformation of PEI [38]. Thus, Hg^2+^ must be optimally coordinated to nitrogen elements of PEI in its N-tetrahedral form at pH 7, leading to the highest sensitivity of the probe [39].

The sensitivity of the PEI-AuNP probe for Hg^2+^ ions was examined as a function of temperature in the range of 30–100°C, and its sensitivity was optimized at 50°C (Figure 5). Also, the sensitivity of PEI-AuNP probe was monitored as a function of NaCl concentration, and it was optimized at 300 mM·NaCl concentration (Figure 5).

The optimum concentration of PEI conjugated to AuNPs was examined in the presence of 0.4 *μ*g·mL^−1^ Hg^2+^ solution, and the absorbance ratio of UV-Vis spectra as a function of PEI concentration revealed that optimum concentration of PEI was ∼33 *μ*M (data not shown).

### 3.5. Quantitation of Hg^2+^, EtHg^+^, MeHg^+^, and PhHg^+^ Using the PEI-AuNP Assay Method

**Figure 6:**
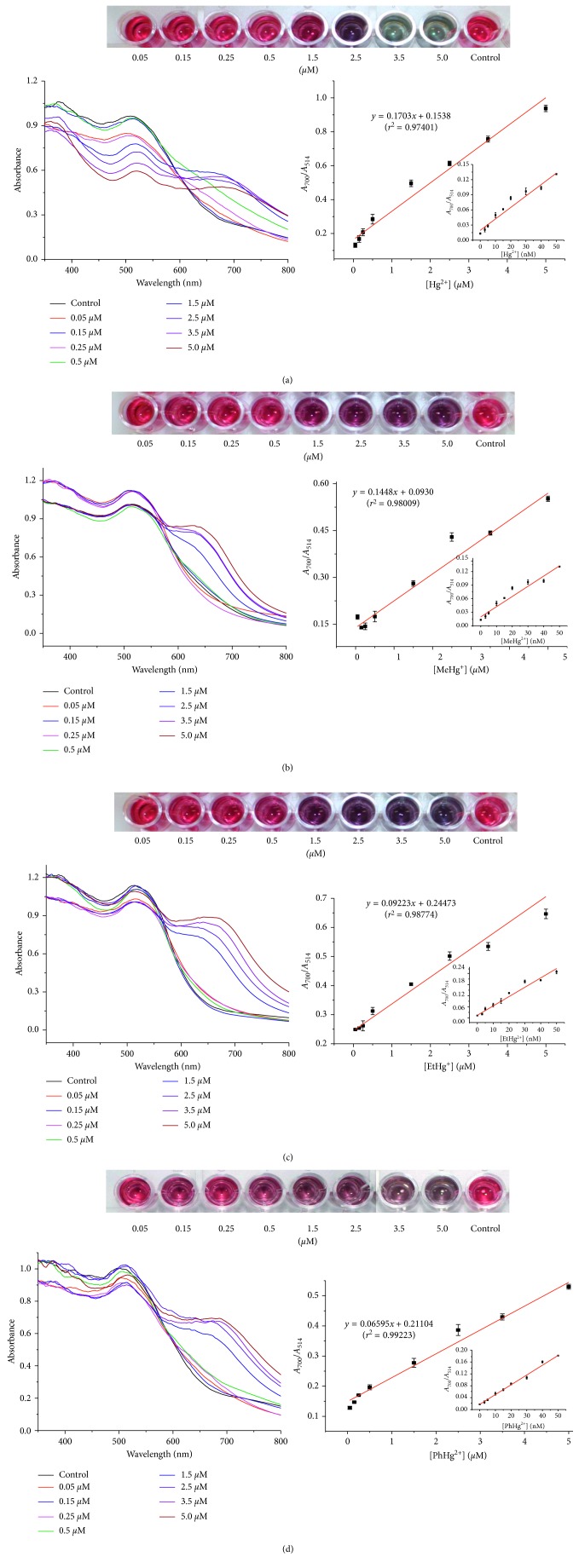
Photographic images, UV-Vis absorption spectra, and absorption ratios (*A*_700_/*A*_514_) of the color change of PEI-AuNPs upon addition of (a) Hg^2+^, (b) MeHg^+^, (c) EtHg^+^, and (d) PhHg^+^ with various concentrations (0.05, 0.15, 0.25, 0.5, 1.5, 2.5, 3.5, and 5.0 *μ*M from left to right) in the presence of 300 mM·NaCl. Inset: plot of *A*_700_/*A*_514_ versus (a) Hg^2+^, (b) MeHg^+^, (c) EtHg^+^, and (d) PhHg^+^ ion concentration (0–50 nM).

The change for color, UV-Vis spectra, and TEM image of the PEI-AuNP probe upon addition of inorganic (Hg^2+^) and organic mercury ions (MeHg^+^, EtHg^+^, and PhHg^+^) were monitored. The color of PEI-AuNPs changed gradually from red to dark violet as the concentration of mercuric ions increased (Figure 6). Also, the absorbance increases at 700 nm and decreases concomitantly at 514 nm, as the concentration of mercuric ions increases (0.05, 0.15, 0.25, 0.50, 1.5, 2.5, 3.5, and 5.0 *μ*M) in PEI-AuNPs solutions. The absorbance ratios for concentrations of each mercuric ion were measured in triplicate. Linear regression analysis of the calibration curve showed a good linearity (*r*^2^ was 0.9817 for Hg^2+^, 0.98009 for MeHg^+^, 0.98774 for EtHg^+^, and 0.99223 for PhHg^+^) within the linear dynamic range of 0.003–3.0 *μ*M. The limits of detection of this probe in tap, pond water, bovine serum, and tuna fish using this probe were measured as 1.72, 1.80, 2.00, and 1.95 nM, respectively, using [3*σ*/slope].

**Figure 7:**
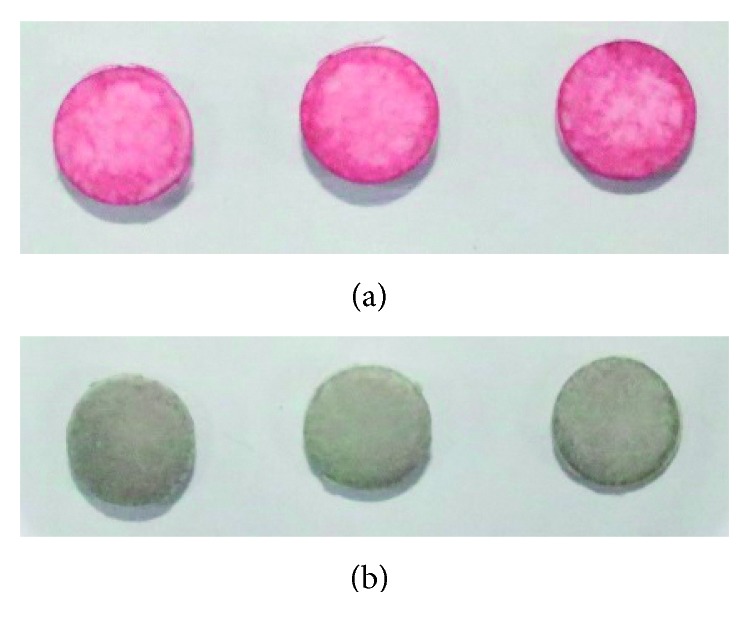
(a) Photographic image of the Whatman paper added with PEI-AuNP solution and dried. (b) Photographic image of the Whatman paper added with 0.1 ppm·Hg^2+^ ion solution.

Paper-type sensor was fabricated, and the present probe solution was dropped to the Whatman paper, and dried it. The color of the Whatman paper turned red (Figure 7), and the functionality of the sensor was tested on water sample containing Hg^2+^ ion. When water sample of 0.1 ppm Hg^2+^ was added onto the Whatman paper disc, its color turned dark purple (Figure 7). This fact showed this paper disc coated with PEI-AuNP solution can be utilized as a paper-type Hg^2+^ sensor.

### 3.6. Application of the PEI-AuNP Probe in the Analyses of Real Samples

To validate the present assay method, the colorimetric responses in real water samples were tested. The tap water, pond water, bovine serum, and tuna fish samples spiked with 0.6 and 1.8 *μ*M·Hg^2+^ were analyzed using the PEI-AuNP probe and DMA. As shown in Table 1, the analytical results of the proposed probe are nearly identical to those obtained using DMA. Hg^2+^ ions in real citrus leaf samples were also determined using both the colorimetric AuNP probe and DMA, as shown in Table 2, and their analytical results are almost the same. Thus, present AuNP-based probe in determination of Hg^2+^ ions seemed to be more advantageous than instrumental methods in terms of simplicity, sensitivity, cost, and time.

The previously reported instrumental methods and nanoparticle assay methods for the detection of Hg^2+^ ions are compared in Table 3, showing that the colorimetric PEI-AuNP probe offers the lowest LOD for the determination of Hg^2+^ ions in aqueous samples.

## 4. Conclusions

A highly sensitive and selective colorimetric probe to determine Hg^2+^ ions was developed using AuNPs conjugated with branched polyethyleneimine. The sensing mechanism of this colorimetric probe was originated from the aggregation of PEI-AuNPs in the presence of Hg^2+^, and the Hg^2+^ ion was found to be selectively coordinated by nitrogen element of PEI conjugated with AuNPs. This method offers simple, highly sensitive, highly selective, and cost-efficient on-site monitoring of the Hg^2+^ ion, allowing the detection of concentrations as low as 1.72 nM to be visually achieved within 40 min.

## Figures and Tables

**Scheme 1:**
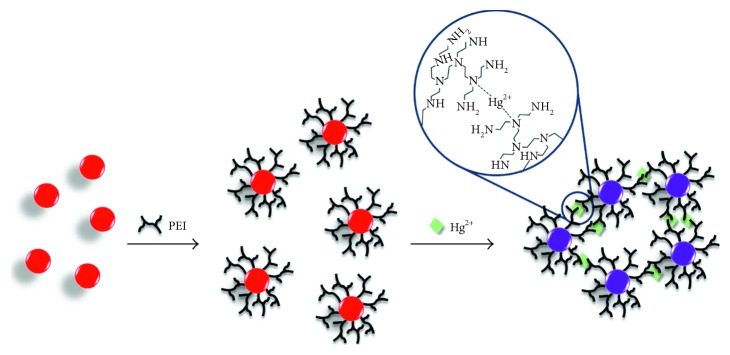
Schematic illustration of the AuNPs capped with PEI, and the aggregation of PEI-AuNPs reacted with Hg^2+^ ion, accompanied by a color change, and the predicted coordination bond between Hg^2+^ ions and PEI-AuNPs.

**Table 1:** Concentrations of Hg^2+^ ions measured by the PEI-AuNP colorimetric probe and DMA in tap water, pond water, tuna fish, and bovine serum samples spiked with Hg^2+^ ions.

Content of Hg^2+^ added to various samples (*n* = 7)						
PEI-AuNP probe						DMA
Sample	Added amount (*μ*M)	Detected amount (*μ*M)	Coefficient of variation (%)	Recovery (%)	LOD (nM)	Detected amount (*μ*M)
Tap water	0.150	0.149 ± 1.53 × 10^−4^	0.102	99.9 ± 0.102	1.72	0.153
	0.500	0.499 ± 0.0101	2.03	99.9 ± 2.03		0.505
Pond water	0.150	0.150 ± 6.92 × 10^−4^	0.460	100.2 ± 0.461	1.80	0.156
	0.500	0.500 ± 3.41 × 10^−3^	0.682	100.1 ± 0.683		0.504
Tuna fish	0.150	0.149 ± 7.09 × 10^−4^	0.473	99.8 ± 0.473	2.00	0.152
	0.500	0.503 ± 0.0155	3.08	100.6 ± 3.10		0.504
Bovine serum	0.150	0.150 ± 6.43 × 10^−4^	0.427	100.1 ± 0.428	1.95	0.155
	0.500	0.503 ± 7.68 × 10^−3^	1.52	100.7 ± 1.53		0.507

**Table 2:** Analytical results for the detection of Hg^2+^ in real citrus leaf samples.

This method					DMA
Samples	Average value (nM)	Recovery (%)	RSD (%)	Accuracy (%)	Average value (nM)
1	22.8 ± 0.34	101.8	1.54	0.22	22.4
2	23.9 ± 0.42	98.7	1.72	1.29	24.3
3	23.1 ± 0.50	98.1	2.13	1.90	23.5

RSD: relative standard deviation.

**Table 3:** Comparison of previously reported instrumental methods and nanoparticle assay methods proposed for the detection of Hg^2+^.

	Sensing principles	Matrices	LOD	Reference
Instruments				
DMA	—	River sediment, bovine liver, tomato leaves, spinach leaves, sewage sludge, mussel tissue, fish tissue, fish protein	1.04 *μ*M	[8]
IC	IC	Tunny fish, oyster, and trumpet	0.5 *μ*M	[9]
HPLC	SPE	Tap water, river water, sea water, and coal-washing waste water	14.9 nM	[10]
HPLC	SAX	Drinking water, lake water, river water, tap water, and sea water	0.8 nM	[11]
Functionalized nanoparticles				
AuNRs	Colorimetric	—	14.9 nM	[12]
CNPs	Fluorometric	Tap water and commercial bottled mineral water	10 nM	[13]
AgNPs	Colorimetric	Tap water	1 nM	[14]
AgNPRs	Colorimetric	Lake water and tap water	3.0 nM	[15]
Lysine–AuNPs	Colorimetric	Tap water	2.9 nM	[16]
DDTC–AuNPs	Colorimetric	Drinking water	2.9 nM	[17]
TCA–AuNCs	Colorimetric	Tap water and lake water	0.5 nM	[18]
PEI–AuNPs	Colorimetric	Tap water and pond water	1.72 nM	This study

SPE: solid phase extraction; SAX: strong anion exchange column; DDTC: diethyldithiocarbamate; TCA: thiocyanuric acid.
